# Liposomal eribulin for advanced adenoid cystic carcinoma, gastric cancer, esophageal cancer, and small cell lung cancer

**DOI:** 10.1002/cam4.4996

**Published:** 2022-07-21

**Authors:** Hibiki Udagawa, Shunji Takahashi, Motohiro Hirao, Makoto Tahara, Satoru Iwasa, Yasuyoshi Sato, Takuya Hamakawa, Kohei Shitara, Hidehito Horinouchi, Keisho Chin, Norikazu Masuda, Takuya Suzuki, Shiori Okumura, Takao Takase, Reiko Nagai, Kan Yonemori

**Affiliations:** ^1^ Department of Thoracic Oncology National Cancer Center Hospital East Kashiwa Japan; ^2^ Department of Medical Oncology The Cancer Institute Hospital of Japanese Foundation for Cancer Research Tokyo Japan; ^3^ Department of Surgery National Hospital Organization Osaka National Hospital Osaka Japan; ^4^ Department of Head and Neck Medical Oncology National Cancer Canter Hospital East Kashiwa Japan; ^5^ Department of Experimental Therapeutics National Cancer Center Hospital Tokyo Japan; ^6^ Department of Gastroenterology and Gastrointestinal Oncology National Cancer Canter Hospital East Kashiwa Japan; ^7^ Department of Thoracic Oncology National Cancer Center Hospital Tokyo Japan; ^8^ Department of Gastroenterology The Cancer Institute Hospital of Japanese Foundation for Cancer Research Tokyo Japan; ^9^ Department of Surgery, Breast Oncology, National Hospital Organization Osaka National Hospital, Osaka, Japan; Current position: Department of Breast and Endocrine Surgery Nagoya University Graduate School of Medicine Nagoya Japan; ^10^ Japan and Asia Clinical Development Department Oncology Business Group, Eisai Co., Ltd. Tokyo Japan; ^11^ Clinical Data Science Department Medicine Development Center, Eisai Co., Ltd. Tokyo Japan; ^12^ Department of Breast and Medical Oncology National Cancer Center Hospital Tokyo Japan

**Keywords:** advanced solid tumors, E7389‐LF, eribulin, granulocyte‐colony stimulating factor, liposome

## Abstract

**Background:**

In this open‐label, Phase 1 study, we explore the safety and efficacy of E7389‐LF (liposomal formulation of eribulin) in Japanese patients with advanced solid tumors.

**Methods:**

This open‐label, Phase 1 study enrolled Japanese adult patients to receive E7389‐LF for the treatment of advanced solid tumors. Treatment with E7389‐LF 2.0 mg/m^2^ every 3 weeks (previously determined maximum tolerated dose) was tested for the treatment of adenoid cystic carcinoma, gastric cancer, esophageal cancer, or small lung cell cancer in the expansion part of this study. Secondary endpoints included safety, objective response rate, best overall response, and progression‐free survival.

**Results:**

As of October 16, 2020, 43 patients were enrolled (adenoid cystic carcinoma, *n* = 12; gastric cancer, *n* = 10; esophageal cancer, *n* = 11; small cell lung cancer, *n* = 10). Thirty‐three patients experienced a Grade ≥3 treatment‐related treatment‐emergent adverse event, most commonly neutropenia (53.5%). Additionally, the incidence of hypersensitivity did not appear to change with a reduced number of infusion steps (2 vs. 4) and patients who were administered prophylactic pegylated granulocyte‐colony stimulating factor had a noticeably lower incidence of Grade 3–4 neutropenia (although this did not have a proper control). The overall objective response rate was 11.6% (95% confidence interval: 3.9–25.1), corresponding to two partial responses in patients with adenoid cystic carcinoma, two partial responses in gastric cancer, and one partial response in esophageal cancer. Median progression‐free survival was longer in the adenoid cystic carcinoma population (16.6 months) than in others.

**Conclusions:**

E7389‐LF 2.0 mg/m^2^ every 3 weeks was well tolerated for the treatment of several different tumor types, and larger studies in these populations are warranted.

## Graphical abstract

This Phase 1 study assessed safety and efficacy of the liposomal formulation of eribulin (E7389‐LF) at a dose of 2.0 mg/m^2^ administered every 3 weeks in 43 patients with advanced adenoid cystic carcinoma (*n* = 12), gastric cancer (*n* = 10), esophageal cancer (*n* = 11), or small cell lung cancer (*n* = 10). Thirty‐three patients (76.7%) experienced a Grade ≥3 treatment‐related treatment‐emergent adverse event (most commonly neutropenia), and 5 patients (11.6%) had an objective response. Changes in the sums of diameters of target lesions from baseline are shown over time in the advanced adenoid cystic carcinoma (A), gastric cancer (B), esophageal cancer (C) and small cell lung cancer (D) cohorts.

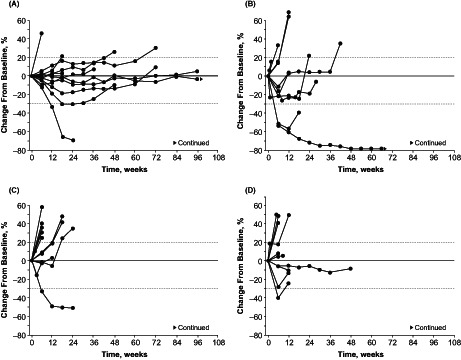

## 1 BACKGROUND

Eribulin is a halichondrin‐class microtubule dynamics inhibitor approved (as eribulin mesylate) for patients with inoperable or recurrent breast cancer or soft tissue sarcoma in Japan.^1^ Moreover, eribulin is approved for the treatment of patients with locally advanced or metastatic breast cancer after ≥1 prior lines of chemotherapy in the European Union^2^, ^3^ and after ≥2 prior lines of chemotherapy in the United States^3^, ^4^ (prior treatments should include a taxane and an anthracycline).

The liposomal formulation of eribulin, E7389‐LF, was created to improve drug delivery into tumors.^5^ Although liposomal drugs are thought to have mechanistic advantages such as reducing drug exposure to healthy tissues and aiding transportation,^6^ the liposome element itself may carry risks for hypersensitivity and infusion reactions.^7^ Previously, a multistep infusion process was used to reduce the risk of hypersensitivity to E7389‐LF in a first‐in‐human study^8^ and in the dose‐escalation part of a Phase 1 study in Japan (Study 114)^9^; however, the complexity of such a process may be seen as inconvenient to patients and medical staff. Furthermore, a risk of neutropenia and febrile neutropenia was previously reported in these studies.^8^, ^9^ Concomitant colony‐stimulating factors (CSFs) may help mitigate this risk, and are recommended by the American Society of Clinical Oncology practice guidelines for patients with a considerable risk of febrile neutropenia (≥20%).^10^ Particularly, pegylated granulocyte CSF (peg‐GCSF) has previously been shown to decrease the incidence of neutropenia and febrile neutropenia in patients receiving chemotherapy for the treatment of solid tumors.^11^, ^12^, ^13^ The extended half‐life of peg‐GCSF also presents the advantage of convenience over non‐peg‐GCSF, which must be administered daily.^13^

The dose‐escalation part of Study 114, a Phase 1 study of E7389‐LF, determined the recommended dosing regimen of E7389‐LF to be 2.0 mg/m^2^ administered once every 3 weeks.^9^ Here, we report results from a dose‐expansion part of this study, which aimed to further evaluate the safety and efficacy of this E7389‐LF dosing regimen in patients with advanced adenoid cystic carcinoma, gastric cancer, esophageal cancer, and small cell lung cancer, as well as evaluating the impact of reducing the number of infusion steps and premedication on hypersensitivity. Additionally, we aimed to determine the effect of preventative peg‐GCSF on the incidence of neutropenia and febrile neutropenia.

## 2 METHODS

Study 114 (ClinicalTrials.gov: NCT03207672) was an open‐label, Phase 1 study conducted at several Japanese clinical sites to evaluate the liposomal formulation of E7389‐LF in patients for the treatment of advanced, nonresectable, or recurrent solid tumors for which no alternative standard therapy or no effective therapy exists. The dose‐expansion portion of this study aims to evaluate the safety and efficacy of E7389‐LF 2.0 mg/m^2^ every 3 weeks, which was determined as the maximum tolerated dose in the dose‐escalation part.^9^ This expansion part included cohorts of patients with nonresectable breast cancer, adenoid cystic carcinoma, gastric cancer, esophageal cancer, and small cell lung cancer—of which the latter 4 cohorts are reported here.

Patients in the adenoid cystic carcinoma cohort were premedicated on cycle 1 day 1 (C1D1) with a steroid and an antihistamine; similar to previous studies,^8^, ^9^ infusion of E7389‐LF then followed a 4‐step process (infusion rate: 0.005 mg/min, 0.01 mg/min, and 0.02 mg/min for ≥10 min each, and then ≤0.2 mg/min). In gastric cancer, esophageal cancer, or small cell lung cancer cohorts, E7389‐LF was infused via a 2‐step process (infusion rate: 0.01 mg/min for ≥10 min, then 0.1 mg/min). For these 3 cohorts, premedication instituted on C1D1 included steroids and antihistamines for the first 10 patients, antihistamines without steroids for the next 10 patients, and no premedication for any subsequently enrolled patients (Figure S1). Patients were also allowed to receive prophylactic or therapeutic peg‐GCSF, if indicated, based on investigator's choice; non‐peg‐GCSF was allowed as an option to treat febrile neutropenia.

### 2.1 Patients

All patients were Japanese and were required to be ≥20 years of age at the time of informed consent. Patients with adenoid cystic carcinoma were required to have nonresectable cancer with confirmed diagnosis and ≥1 prior chemotherapy regimen (unless contraindicated). In the three other cohorts described herein (i.e., gastric cancer, esophageal cancer, and small cell lung cancer) eligible patients were required to have nonresectable cancer with confirmed diagnosis and ≥2 prior chemotherapy regimens (unless contraindicated); patients with esophageal cancer were enrolled with ≥1 prior chemotherapy regimen if they received a combination regimen including a platinum‐based therapy and a taxane. Patients in all four cohorts were required to have an Eastern Cooperative Oncology Group performance status (ECOG PS) of 0–1; ≥1 lesion measurable by RECIST version 1.1; and adequate organ function. Additionally, patients who had had prior anticancer regimens were required to have an adequate washout period before study drug administration (≥3 weeks for chemotherapy, hormonal therapy, and radiotherapy; ≥4 weeks for antibody therapy or investigational drug/device; ≥2 weeks for blood/platelet transfusion or any GCSF), and adverse events due to previous anticancer therapy should have returned to either Grade 0 or 1 severity, except for alopecia and Grade 2 peripheral neuropathy.

Key exclusion criteria included cardiac conditions such as a New York Heart Association heart failure class II or above; unstable ischemic heart disease (myocardial infarction within 6 months prior to starting study drug, or angina requiring use of nitrates more than once weekly, or prolongation of corrected QT interval to >480 ms); any hypersensitivity reaction to a liposomal formulation agent; or previous treatment with eribulin.

### 2.2 Outcomes

The primary endpoint of Study 114 was to determine the maximum tolerated dose of E7389‐LF (determined previously^9^). Secondary endpoints included safety assessments, objective response rate defined by best overall response, and progression‐free survival. Exploratory endpoints included disease control rate and clinical benefit rate defined by best overall response.

### 2.3 Statistical methods

A sample size of 10 patients per cohort was planned for the adenoid cystic carcinoma, gastric cancer, esophageal cancer, and small cell lung cancer tumor types. All adverse events were graded via Common Terminology Criteria for Adverse Events v4.03 and listed by Medical Dictionary for Regulatory Activities preferred term. All efficacy and safety analyses were performed on all patients who received ≥1 dose of the study drug. Incidences of Grade ≥3 neutropenia and febrile neutropenia were assessed by baseline neutrophil count, using a cutoff of 3000 neutrophils/mm^3^ at baseline—the value at which differences were seen in the incidences of Grade 3–4 febrile neutropenia and neutropenia. Tumor assessments were performed every 6 weeks by the investigator based on RECIST version 1.1. The best overall response was summarized and objective response rate (defined as the proportion of patients with a complete response or partial response), disease‐control rate (defined as the proportion of patients with a complete response, partial response, or stable disease ≥5 weeks after C1D1), and clinical benefit rate (defined as the proportion of patients with a complete response, partial response, or stable disease lasting for ≥23 weeks) will be reported with 95% confidence interval (CI) by cohort. Median progression‐free survival will be provided with 95% CI. Statistical analyses were performed using SAS software (version 9.2 or later).

## 3 RESULTS

### 3.1 Patients

As of October 16, 2020, 43 patients had been enrolled and treated (adenoid cystic carcinoma, *n* = 12; gastric cancer, *n* = 10; esophageal cancer, *n* = 11 [all had squamous cell carcinoma]; small cell lung cancer, *n* = 10; Table 1). More than 10 patients were enrolled in the cohorts mentioned above for operational reasons—the three additional patients were enrolled as they had signed informed consent forms while enrollment was ending. Analyses were conducted prior to database lock. Overall, the median patient age was 60.0 years (range 46–77) and most patients (67.4%) had an ECOG PS of 0. The median number of prior anticancer therapy regimens was 3 (range 1–6). By the data cutoff date, 33 patients (76.7%) completed treatment and 3 (7%) had treatment ongoing. Seven patients (16.3%) discontinued treatment, of whom two discontinued due to adverse events and five discontinued due to patient choice.

**TABLE 1:** Baseline patient demographics

Characteristic	Cohort				Overall *N* = 43
	ACC *n* = 12	GC *n* = 10	EGC^a^ *n* = 11	SCLC *n* = 10	
Median age, years (range)	55.5 (48–68)	61.5 (46–77)	60.0 (47–77)	66.0 (48–75)	60.0 (46–77)
Sex, *n* (%)					
Male	5 (41.7)	7 (70.0)	9 (81.8)	7 (70.0)	28 (65.1)
Female	7 (58.3)	3 (30.0)	2 (18.2)	3 (30.0)	15 (34.9)
ECOG PS, *n* (%)					
0	11 (91.7)	6 (60.0)	8 (72.7)	4 (40.0)	29 (67.4)
1	1 (8.3)	4 (40.0)	3 (27.3)	6 (60.0)	14 (32.6)
Site of primary lesion for solid tumor, *n* (%)					
Bronchus	0	0	0	2 (20.0)	2 (4.7)
Esophagus	0	0	11 (100)	0	11 (25.6)
Floor of mouth	2 (16.7)	0	0	0	2 (4.7)
Gastric	0	8 (80.0)	0	0	8 (18.6)
Gastro‐esophageal junction	0	2 (20.0)	0	0	2 (4.7)
Gums	2 (16.7)	0	0	0	2 (4.7)
Lung	0	0	0	8 (80.0)	8 (18.6)
Thymus gland	1 (8.3)	0	0	0	1 (2.3)
Tongue	2 (16.7)	0	0	0	2 (4.7)
Other	5 (41.7)	0	0	0	5 (11.6)
Median number of prior anticancer regimens, *n* (range)	1 (1–6)	5 (2–6)	3 (2–6)	3 (2–4)	3 (1–6)
Patients who received prior anticancer therapies^b^, *n* (%)	12 (100)	10 (100)	11 (100)	10 (100)	43 (100)
Targeted therapies					
Cetuximab	3 (25.0)	0	0	0	3 (7.0)
Ramucirumab	0	10 (100)	0	0	10 (23.3)
Trastuzumab	0	3 (30.0)	0	0	3 (7.0)
Immune checkpoint inhibitors					
Durvalumab	0	0	0	2 (20.0)	2 (4.7)
Nivolumab	2 (16.7)	9 (90.0)	1 (9.1)	0	12 (27.9)
Pembrolizumab	0	0	0	2 (20.0)	2 (4.7)
Chemotherapies					
Amrubicin	0	0	0	9 (90.0)	9 (20.9)
Capecitabine	0	2 (20.0)	0	0	2 (4.7)
Carboplatin	5 (41.7)	0	1 (9.1)	6 (60.0)	12 (27.9)
Cisplatin	3 (25.0)	3 (30.0)	10 (90.9)	7 (70.0)	23 (53.0)
Docetaxel	0	2 (20.0)	5 (45.5)	0	7 (16.3)
Etoposide	0	0	0	10 (100)	10 (23.3)
Fluorouracil	2 (16.7)	1 (10.0)	11 (100)	0	14 (32.6)
Gimeracil, oteracil, tegafur	5 (41.7)	10 (100)	3 (27.3)	0	18 (41.9)
Irinotecan	0	5 (50.0)	0	4 (40.0)	9 (20.9)
Oxaliplatin	0	6 (60.0)	0	0	6 (14.0)
Paclitaxel	6 (50.0)	10 (100)	9 (81.8)	0	25 (58.1)
Other					
Investigational drug	1 (8.3)	3 (30.0)	2 (18.2)	0	6 (14.0)

Abbreviations: ACC, adenoid cystic carcinoma; ECOG PS, Eastern Cooperative Oncology Group performance status; EGC, esophageal cancer; GC, gastric cancer; SCLC, small cell lung cancer.

^a^ All patients with EGC had squamous cell carcinoma; ^b^therapies are listed if they were received in ≥20% of any cohort.

### 3.2 Safety

In the overall patient population, all 43 patients experienced treatment‐related treatment‐emergent adverse events (TEAEs) and 33 patients (76.7%) experienced at least one treatment‐related TEAE of Grade ≥3 severity (Table S1). Overall, nine patients (20.9%) had at least one treatment‐related serious TEAE. Twenty‐two patients (51.2%) had treatment‐related TEAEs leading to dose reduction (most commonly neutropenia); two patients (4.7%) discontinued treatment due to treatment‐related TEAEs (Grade 3 pneumonia not due to interstitial lung disease, *n* = 1; Grade 3 hypersensitivity reaction, *n* = 1). The most common Grade 3–5 treatment‐related TEAEs were neutropenia (53.5%), leukopenia (34.9%), and febrile neutropenia (11.6%) (Table 2). One death occurred due to a TEAE (general physical health deterioration) in the small cell lung cancer cohort; however, it was considered not related to treatment.

**TABLE 2:** Treatment‐related TEAEs in ≥10% of all patients

Treatment‐related TEAEs, *n* (%)	Overall population (*N* = 43); severity of event		
	Any grade^a^	Grade 3	Grade 4
Hematologic events			
Leukopenia	24 (55.8)	12 (27.9)	3 (7.0)
Neutropenia	24 (55.8)	7 (16.3)	16 (37.2)
Thrombocytopenia	23 (53.5)	0	0
Anemia	7 (16.3)	2 (4.7)	0
Febrile neutropenia	5 (11.6)	5 (11.6)	0
Nonhematologic events			
Aspartate aminotransferase increased	22 (51.2)	2 (4.7)	0
Decreased appetite	20 (46.5)	1 (2.3)	0
Stomatitis	19 (44.2)	0	0
Alanine aminotransferase increased	16 (37.2)	3 (7.0)	0
Alopecia	16 (37.2)	0	0
Nausea	14 (32.6)	1 (2.3)	0
Peripheral sensory neuropathy	13 (30.2)	0	0
Fatigue	12 (27.9)	0	0
Pyrexia	11 (25.6)	0	0
Gamma‐glutamyl transferase increased	10 (23.3)	2 (4.7)	0
Rash, maculopapular	9 (20.9)	1 (2.3)	0
Blood alkaline phosphatase increased	7 (16.3)	0	0
Drug hypersensitivity	6 (14.0)	1 (2.3)	0
Dysgeusia	6 (14.0)	0	0
Malaise	6 (14.0)	0	0
Rash	6 (14.0)	0	0
Constipation	5 (11.6)	0	0

TEAE, treatment‐emergent adverse event.

^a^ No Grade 5 treatment‐related TEAEs occurred.

Five patients (11.6%) experienced hypersensitivity reactions in cycle 1 and this occurred in both the 2‐step and 4‐step infusion arms, but there were no occurrences of Grade ≥3 severity (Table 3). Of note, there were no marked differences in the incidences of Grade 1 or 2 hypersensitivity reactions across the 4 cohorts, which varied in infusion rates and number of premedications.

**TABLE 3:** Adverse events of special interest during cycle 1

Characteristic	ACC Cohort	GC, EGC, and SCLC Cohorts^a^		
Infusion process	4 step^b^	2 step^c^	2 step^c^	2 step^c^
Premedication	Steroid + antihistamine	Steroid + antihistamine	Antihistamine	None
*n*	12	11	10	10
Patients with any hypersensitivity reaction, *n* (%)	1 (8.3)	0	2 (20.0)	2 (20.0)
Grade 1	1 (8.3)	0	1 (10.0)	0
Grade 2	0	0	1 (10.0)	2 (20.0)
Grade 3	0	0	0	0
Grade 4	0	0	0	0

Abbreviations: ACC, adenoid cystic carcinoma; EGC, esophageal carcinoma; GC, gastric carcinoma; SCLC, small cell lung cancer.

^a^ The first 10 patients enrolled in the GC, EGC, and SCLC cohorts were to receive steroid + antihistamine premedication; the next 10 patients enrolled were to receive only antihistamine premedication; the remaining patients enrolled were not to receive any premedication, regardless of tumor type.

^b^ The 4‐step E7389‐LF infusion rate: 0.005 mg/min, 0.01 mg/min, and 0.02 mg/min for ≥10 min each, followed by ≤0.2 mg/min.

^c^ The 2‐step E7389‐LF infusion rate: 0.01 mg/min for ≥10 min, followed by 0.1 mg/min.

In Cycle 1, rates of Grade 3–4 neutropenia and febrile neutropenia were lower in patients who received prophylactic peg‐GCSF (Table 4) and were similarly reduced regardless of sex. Depending on baseline neutrophil counts (≥3000 cells/mm^3^ vs. <3000 cells/mm^3^), Grade 3–4 neutropenia occurred in 70.0% versus 100%, respectively, of patients who did not receive prophylactic peg‐GCSF therapy compared with 5.6% versus 22.2%, respectively, of patients who did (Table S2).

**TABLE 4:** Incidences of Grade 3 and/or 4 neutropenia and Grade 3 and/or 4 febrile neutropenia with or without prior prophylactic peg‐GCSF treatment during cycle 1

MedDRA preferred term, *n* (%)	With prophylactic peg‐GCSF *n* = 27	Without prophylactic peg‐GCSF *n* = 16
Neutropenia (Grade 3 + 4) ^a^	3 (11.1)	13 (81.3)
Grade 3	2 (7.4)	1 (6.3)
Grade 4	1 (3.7)	12 (75.0)
Febrile neutropenia (Grade 3 + 4) ^b^	1 (3.7)	2 (12.5)
Grade 3	1 (3.7)	2 (12.5)
Grade 4	0	0

Abbreviations: MedDRA, Medical Dictionary for Regulatory Activities; peg‐GCSF, pegylated granulocyte colony‐stimulating factor.

^a^ Of the 28 male patients, 9 (32.1%) had Grade 3 and/or 4 neutropenia during Cycle 1—of whom 1 (5.6%) had received prophylactic peg‐GCSF; of the 15 female patients, 7 (46.7%) had Grade 3 and/or 4 neutropenia—of whom 2 (22.2%) had received prior peg‐GCSF.

^b^ Of the 28 male patients, 2 (7.1%) had Grade 3 and/or 4 febrile neutropenia during cycle 1—of whom 1 (5.6%) had received prophylactic peg‐GCSF; of the 15 female patients, 1 (6.7%) had Grade 3 and/or 4 febrile neutropenia—this patient had not received prior peg‐GCSF.

### 3.3 Efficacy

**FIGURE 1:**
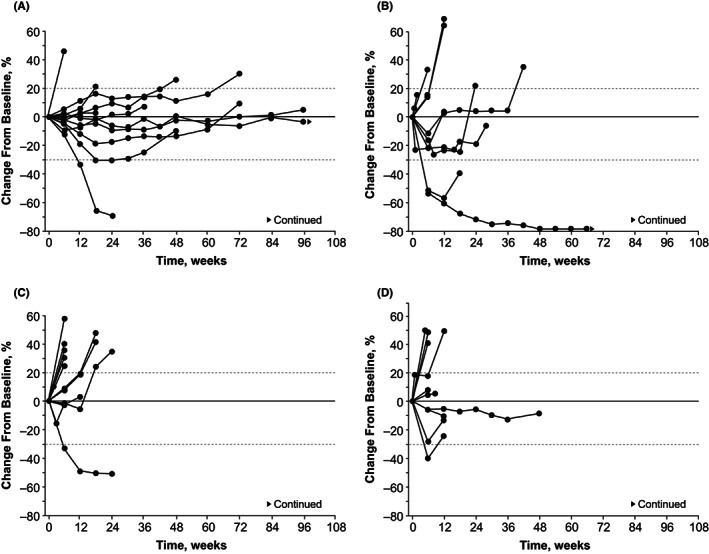
Percentage changes from baseline in the sums of diameters of target lesions over time in the adenoid cystic carcinoma cohort (A), gastric cancer cohort (B), esophageal cancer cohort (C), and small cell lung cancer cohort (D)

The objective response rate among all patients was 11.6% (95% CI: 3.9–25.1; Table 5), corresponding with 5 patients who achieved a partial response (adenoid cystic carcinoma, *n* = 2; gastric cancer, *n* = 2; esophageal cancer, *n* = 1). Additionally, 26 patients experienced stable disease (adenoid cystic carcinoma, *n* = 9; gastric cancer, *n* = 6; esophageal cancer, *n* = 5; small cell lung cancer, *n* = 6), resulting in disease‐control rates per cohort as follows: adenoid cystic carcinoma, 91.7%; gastric cancer, 80.0%; esophageal cancer, 54.5%; and small cell lung cancer, 60.0%. The median progression‐free survival was 16.6 months in the adenoid cystic carcinoma cohort compared with 3.5, 2.8, and 2.4 months in the gastric cancer, esophageal cancer, and small cell lung cancer cohorts, respectively. The percentage changes in the sums of diameters of target lesions over time, per cohort, are shown in Figure 1, and the maximum percentage changes from baseline in the sums of diameters of target lesions for all cohorts are shown in Figure S2.

**TABLE 5:** Efficacy outcomes

Characteristics	Cohort				Overall *N* = 43
	ACC *n* = 12	GC *n* = 10	EGC *n* = 11	SCLC *n* = 10	
BOR, *n* (%)					
PR	2 (16.7)	2 (20.0)	1 (9.1)	0	5 (11.6)
SD	9 (75.0)	6 (60.0)	5 (45.5)	6 (60.0)	26 (60.5)
PD	1 (8.3)	2 (20.0)	5 (45.5)	4 (40.0)	12 (27.9)
ORR^a^, *n* (%)	2 (16.7)	2 (20.0)	1 (9.1)	0	5 (11.6)
95% CI^b^	2.1–48.4	2.5–55.6	0.2–41.3	0.0–30.8	3.9–25.1
DCR^c^, *n* (%)	11 (91.7)	8 (80.0)	6 (54.5)	6 (60.0)	31 (72.1)
95% CI^b^	61.5–99.8	44.4–97.5	23.4–83.3	26.2–87.8	56.3–84.7
CBR^d^, *n* (%)	8 (66.7)	5 (50.0)	2 (18.2)	1 (10.0)	16 (37.2)
95% CI^b^	34.9–90.1	18.7–81.3	2.3–51.8	0.3–44.5	23.0–53.3
Durable SD rate^**e**^, *n* (%)	6 (50.0)	3 (30.0)	1 (9.1)	1 (10.0)	11 (25.6)
95% CI^b^	21.1–78.9	6.7–65.2	0.2–41.3	0.3–44.5	13.5–41.2
Median PFS^f^, months (95% CI)	16.6 (4.2–NE)	3.5 (0.4–6.6)	2.8 (1.2–4.4)	2.4 (1.0–2.9)	4.2 (2.6–5.4)
Quartile 1 (95% CI)	11.1 (1.2–16.6)	2.6 (0.4–4.3)	1.2 (1.2–2.8)	1.3 (1.0–2.6)	1.4 (1.2–2.7)
Quartile 3 (95% CI)	NE (11.3–NE)	6.6 (2.8–NE)	4.4 (1.4–5.4)	2.9 (2.3–11.1)	11.1 (5.3–16.8)

Abbreviations: ACC, adenoid cystic carcinoma; BOR, best overall response; CBR, clinical benefit rate; CI, confidence interval; CR, complete response; DCR, disease control rate; EGC, esophageal cancer; GC, gastric cancer; NE, not estimable; ORR, objective response rate; PD, progressive disease; PFS, progression‐free survival; PR, partial response; SCLC, small cell lung cancer; SD, stable disease.

^a^ Proportion of patients with CR + PR.

^b^ Calculated using the Clopper‐Pearson method.

^c^ Proportion of patients with CR + PR + SD (SD ≥5 weeks).

^d^ CR + PR + durable SD (SD ≥23 weeks).

^e^ Proportion of patients with duration of SD ≥23 weeks.

^f^ Calculated using Kaplan–Meier estimates and Greenwood Formula.

## 4 DISCUSSION

In this Phase 1 study‐expansion part, E7389‐LF 2.0 mg/m^2^ given every 3 weeks had a tolerable and manageable safety profile in patients who had been pretreated for adenoid cystic carcinoma, gastric cancer, esophageal cancer, or small cell lung cancer.

The treatment‐related TEAEs observed in this expansion part were similar to the TEAEs of any cause in the dose‐escalation part of this study,^9^ acknowledging limitations in comparing groups with different causality. Patients who received prophylactic treatment with peg‐GCSF appeared to experience lower rates of Grade 3–4 neutropenia (11.1%) and Grade 3 febrile neutropenia (3.7%) versus patients who did not receive prophylactic treatment (81.3% and 12.5%, respectively). It is important to note that the necessity of administering peg‐GCSF was determined by the investigator, and rates of neutropenia in patients who did or did not receive peg‐GCSF were not compared by randomization. Moreover, the incidence of Grade 3–4 neutropenia and febrile neutropenia also appeared to be lower in patients with neutrophil levels ≥3000 cells/mm^3^ at baseline, although the small sample size limits this interpretation. The incidence of hypersensitivity reactions was low and did not appear to change with a reduced number of infusion steps or a reduced number of premedication therapies. This suggests that the 2‐step rate (a more convenient method than the previously used 4‐step rate) was acceptable. Considering that the incidence of hypersensitivity did not appear to change with type of premedication, further investigation might be needed to determine the necessity of steroid and/or antihistamine agents.

Partial responses were observed in patients in the pretreated adenoid cystic carcinoma (*n* = 2), heavily pretreated gastric cancer (*n* = 2) and esophageal cancer (*n* = 1) groups, and a minority of patients overall had a best overall response of progressive disease. The objective response rate of 20% (2/10 responses) in patients with pretreated gastric cancer compares favorably to Phase 3 studies of approved medications (4% for TAS‐102 and 11% for nivolumab),^14^, ^15^ implying that E7389‐LF may have efficacy for heavily pretreated gastric cancer. With a median progression‐free survival of 16.6 months in the adenoid cystic carcinoma cohort, this duration was notably higher than other cohorts in this study (2.4–3.5 months), despite having a similar objective response rate. Acknowledging the limitations of cross‐study comparisons, this duration was also noticeably longer than that in a previous report of eribulin mesylate for recurrent/metastatic adenoid cystic carcinoma (progression‐free survival, 3.5 months).^16^ The median progression‐free survival of the adenoid cystic carcinoma cohort was comparable to that from a Phase 2 study of lenvatinib in patients with progressive, recurrent, or metastatic adenoid cystic carcinoma (17.5 months).^17^ Compared with other cohorts, a greater proportion of patients in the adenoid cystic carcinoma cohort had stable disease for at least 23 weeks, implying that less disease progression was seen in this group, regardless of best overall response. Considering these unprecedented results, patients in this group may have had less‐severe disease on average. Notably, the median number of lines of prior treatment was lower in this group^1^ compared with the other cohorts,^3^, ^4^, ^5^ and a greater proportion of patients had an ECOG PS of 0 (91.7% vs. 40.0–72.7%). Generally, efficacy was promising across cohorts as indicated by generally high disease‐control rates, and the median PFS was similar among the gastric cancer, esophageal cancer, and small cell lung cancer cohorts.

One limitation to note is the small sample size, particularly per cohort. Although 43 patients were enrolled overall, around 10 were enrolled per tumor type cohort, which ultimately limits the efficacy conclusions that can be drawn. Another limitation is that, to ensure data from this Phase 1 study were available to support further clinical development of E7389‐LF, analyses were performed before database lock.

In summary, E7389‐LF was well‐tolerated for the treatment of several different tumor types, and larger studies in these populations are warranted. In terms of efficacy, partial responses were observed in several tumor types. Peg‐GCSF may be an effective option to prevent neutropenia and febrile neutropenia in specific patients treated with E7389‐LF. Infusion of E7389‐LF with a 2‐step process was tolerable. The necessity of premedication prior to E7389‐LF therapy remains unclear, and further investigation is warranted.

## AUTHORS' CONTRIBUTIONS

Study design and conceptualization: Takuya Suzuki.

Data collection: Hibiki Udagawa, Shunji Takahashi, Motohiro Hirao, Makoto Tahara, Satoru Iwasa, Yasuyoshi Sato, Takuya Hamakawa, Kohei Shitara, Hidehito Horinouchi, Keisho Chin, Norikazu Masuda, and Kan Yonemori.

All authors contributed to data analysis, data interpretation, and drafting of the manuscript, and gave final approval for publishing.

## FUNDING INFORMATION

This work was supported by Eisai Co., Ltd., Tokyo, Japan. No specific grant number applies.

Medical writing support was provided by Michael Venditto, PharmD, of Oxford PharmaGenesis Inc., Newtown, PA, USA. This support was funded by Eisai Inc., Nutley, NJ, USA.

## CONFLICT OF INTEREST

**H Udagawa**, **M Hirao**, and **T Hamakawa** have nothing to declare.

**S Takahashi** reports receiving grants from Eisai during the conduct of the study, as well as grants and personal fees from Novartis, Taiho, Chugai, Bayer, and Daiichi‐Sankyo, and grants from AstraZeneca outside the submitted work.

**M Tahara** reports receiving grants and personal fees from Eisai during the conduct of the study, as well as grants and personal fees from Ono Pharmaceutical, MSD, Bayer, BMS, Pfizer, Novartis, Lilly, Amgen, and Rakuten Medical, and personal fees from Merck Biopharma, LOXO and Celgene outside the submitted work.

**S Iwasa** reports receiving research funding fees from Eisai Inc., for activities pertaining to or outside the submitted work, as well as research funding from Astellas, Bayer, Bristol Myers Squibb, Daiichi‐Sankyo, Merck Biopharma, Ono Pharmaceutical, and Pfizer, and honoraria from Bristol Myers Squibb, Chugai, Eli Lilly, Ono Pharmaceutical, and Taiho, unrelated to the submitted work.

**Y Sato** reports receiving honoraria from ONO Pharmaceutical Co., Ltd, Bristol Myers Squibb, MSD KK, and TAIHO Pharmaceutical Co., Ltd, unrelated to the submitted work.

**K Shitara** reports receiving paid consulting or advisory roles for Astellas, Lilly, Bristol Myers Squibb, Takeda, Pfizer, Ono, MSD, Taiho, Novartis, AbbVie, GlaxoSmithKline, Daiichi Sankyo, Amgen, and Boehringer Ingelheim, honoraria from Novartis, AbbVie, and Yakult, and research funding from Astellas, Lilly, Ono, Sumoitomo Dainippon, Daiichi Sankyo, Taiho, Chugai, MSD, Medi Science, and Eisai.

**H Horinouchi** reports grants from Eisai during the conduct of the study, grants and personal fees from MSD, personal fees from Lilly, grants and personal fees from AstraZeneca, grants and personal fees from BMS, grants and personal fees from Ono, personal fees from Merck Biopharma, grants from Daiichi‐Sankyo, grants and personal fees from Janssen, grants from Genomic health, grants and personal fees from Chugai, personal fees from Kyowa‐Kirin, personal fees from Nihonkayaku, grants and personal fees from Abbvie, outside the submitted work.

**K Chin** reports receiving honoraria from Bristol Myers Squibb, Chugai Pharmaceutical Co., Ltd., Ono Pharmaceutical Co., Ltd., and Taiho Pharma, unrelated to the submitted work.

**N Masuda** reports receiving honoraria (eg, lecture fees) from Chugai, AstraZeneca, Pfizer, Eli‐Lilly, and Eisai, research funding from Chugai, AstraZeneca, Kyowa‐Kirin, MSD, Novartis, Pfizer, Eli‐Lilly, Eisai, Sanofi, Nihon‐Kayaku and Daiichi Sankyo, and a position on the board of directors from the Japanese Breast Cancer Society (JBCS) and Japan Breast Cancer Research Group Association (JBCRG).

**T Suzuki**, **S Okumura**, **T Takase**, and **R Nagai** are employees of Eisai Co., Ltd., and have nothing else to disclose.

**K Yonemori** reports receiving a grant from Eisai Inc. for trial management related to the submitted work, as well as honoraria from Pfizer, Eisai, AstraZeneca, Eli Lilly, Takeda, Chugai, Fuji Film Pharma, a consultancy or advisory role from Novartis, Eisai, AstraZeneca, Chugai, Takeda, Genmab, and OncXerna, and research support (to institution) from MSD, Daiichi‐Sankyo, AstraZeneca, Taiho, Pfizer, Novartis, Takeda, Chugai, Ono, Sanofi, Seattle genetics, Eisai, Eli Lilly, Genmab, Boehringer Ingelheim, Kyowa Hakko Kirin, Nihon Kayaku, Seagen, and Haihe, unrelated to the submitted work.

## ETHICS APPROVAL AND CONSENT TO PARTICIPATE

This study was conducted in accordance with principles of the World Medical Association Declaration of Helsinki, all applicable Japanese Good Clinical Practice guidelines and regulations, and Article 14, Paragraph 3, and Article 80–2 of the Pharmaceutical Affairs Law (Law No. 145, 1960) for studies conducted in Japan.

Ethical approval and written informed consent were granted and approved by the local institutional review board for each study site; signed informed consent forms were obtained from each patient prior to enrollment.

This study was registered at ClinicalTrials.gov with an identifier of NCT03207672.

## CONSENT FOR PUBLICATION

Personal patient data are not included in this manuscript. All authors confirm their consent for this article to be published.

## Supporting information

Supinfo

## Data Availability

The data will not be available for sharing at this time because the data are commercially confidential. However, Eisai Inc., will consider written requests to share the data on a case‐by‐case basis.
