# Supplementary material for: Liposomal eribulin for advanced adenoid cystic carcinoma, gastric cancer, esophageal cancer, and small cell lung cancer
Source: Cancer Med. 2022 Jul 21;12(2):1269–78. doi: 10.1002/cam4.4996 (PMC9883537; doi:10.1002/cam4.4996)
Supplement: Supplementary file 1 — Supinfo [file CAM4-12-1269-s001.docx]

***Supplemental Figures/Tables***

**Table S1. Safety Summary**

| **Characteristics, n (%)** | **Cohort** | | | | **Overall  N = 43** |
| --- | --- | --- | --- | --- | --- |
|  | **ACC  n = 12** | **GC  n = 10** | **EGC**  **n = 11** | **SCLC**  **n = 10** |  |
| **Treatment-related TEAE** | 12 (100) | 10 (100) | 11 (100) | 10 (100) | 43 (100) |
| Grade 3–4^a^ | 10 (83.3) | 7 (70.7) | 8 (72.2) | 8 (80.0) | 33 (76.7) |
| **Treatment-related SAE** | 4 (33.3) | 2 (20.0) | 1 (9.1) | 2 (20.0) | 9 (20.9) |
| **Treatment-related TEAEs leading to dose adjustment** | | | | | |
| Dose reduction | 10 (83.3) | 4 (40.0) | 3 (27.3) | 5 (50.0) | 22 (51.2) |
| Drug discontinuation | 2 (16.7) | 0 | 0 | 0 | 2 (4.7) |

^a^No grade 5 treatment-related TEAEs occurred.

ACC, adenoid cystic carcinoma; EGC, esophageal cancer; GC, gastric cancer; SAE, serious adverse event; SCLC, small cell lung cancer; TEAE, treatment-emergent adverse event.

**Table S2. Incidences of Grade 3 and/or 4 Neutropenia and Grade 3 and/or 4 Febrile Neutropenia by Prior Prophylactic Peg-GCSF Treatment Category and by Baseline Neutrophil Count During Cycle 1**

| **MedDRA Preferred Term, n (%)** | **Neutrophils ≥ 3000 cells/mm^3^ at Baseline** | | | **Neutrophils < 3000 cells/mm^3^ at Baseline** | | |
| --- | --- | --- | --- | --- | --- | --- |
|  | **Prophylactic  Peg-GCSF**  **n = 18** | **No Prophylactic Peg-GCSF**  **n = 10** | **Overall**  **n = 28** | **Prophylactic Peg-GCSF**  **n = 9** | **No Prophylactic Peg-GCSF**  **n = 6** | **Overall**  **n = 15** |
| **Neutropenia (grade 3 + 4)** | 1 (5.6) | 7 (70.0) | 8 (28.6) | 2 (22.2) | 6 (100) | 8 (53.3) |
| Grade 3 | 1 (5.6) | 1 (10.0) | 2 (7.1) | 1 (11.1) | 0 | 1 (6.7) |
| Grade 4 | 0 | 6 (60.0) | 6 (21.4) | 1 (11.1) | 6 (100) | 7 (46.7) |
| **Febrile neutropenia (grade 3 + 4)** | 0 | 1 (10.0) | 1 (3.6) | 1 (11.1) | 1 (16.7) | 2 (13.3) |
| Grade 3 | 0 | 1 (10.0) | 1 (3.6) | 1 (11.1) | 1 (16.7) | 2 (13.3) |
| Grade 4 | 0 | 0 | 0 | 0 | 0 | 0 |

MedDRA, Medical Dictionary for Regulatory Activities; peg-GCSF, pegylated granulocyte colony-stimulating factor.

**Figure S1. Study Design**

**
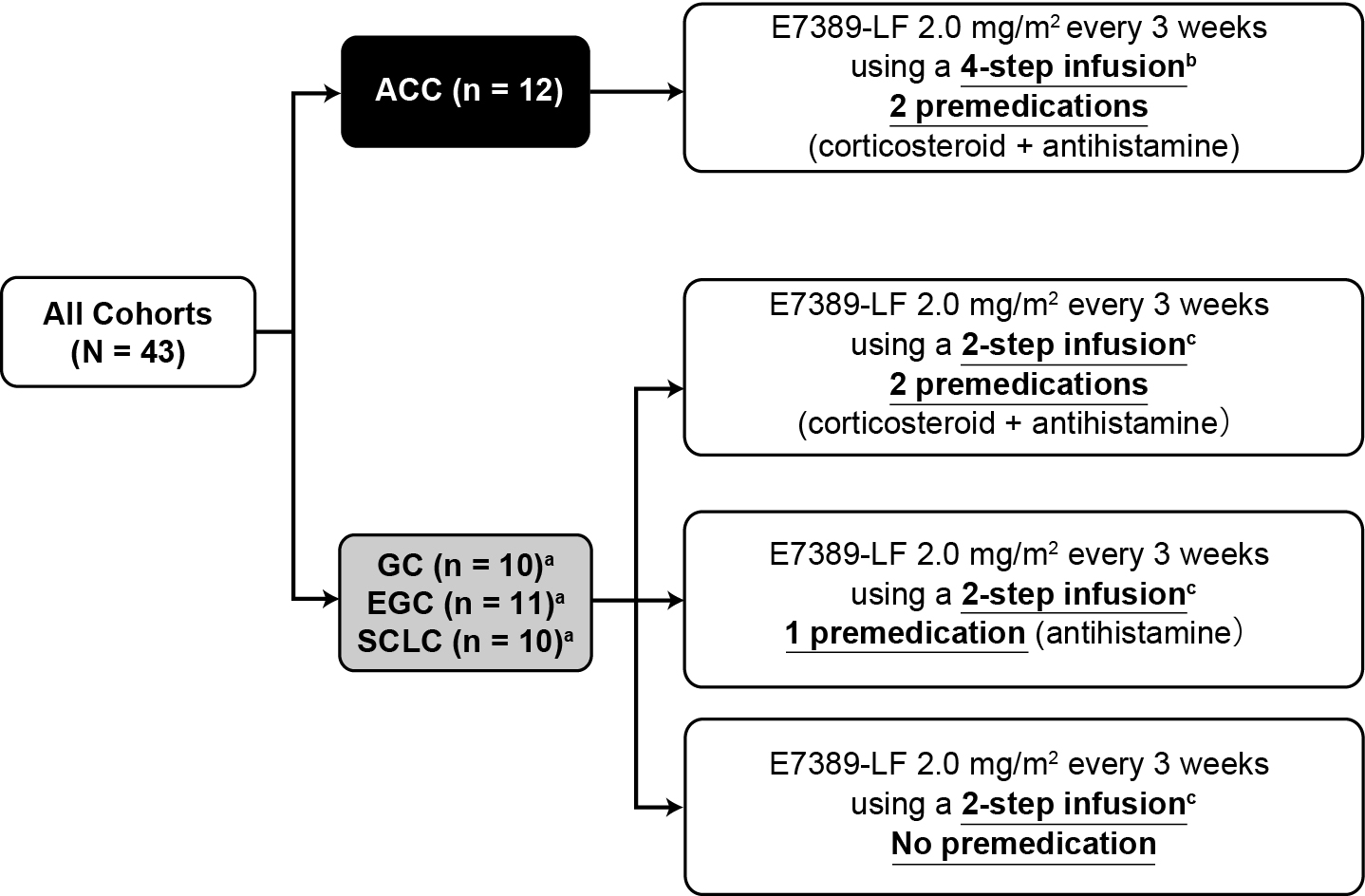
**

^a^The first 10 patients enrolled in the GC, EGC, and SCLC cohorts were to receive steroid and antihistamine premedication; the next 10 patients enrolled were to receive only antihistamine premedication; the remaining patients enrolled were not to receive any premedication, regardless of tumor type; ^b^the 4-step E7389-LF infusion rate: 0.005 mg/min, 0.01 mg/min, and 0.02 mg/min for ≥10 min each, followed by ≤0.2 mg/min; ^c^the 2-step E7389-LF infusion rate: 0.01 mg/min for ≥10 min, followed by 0.1 mg/min.

ACC, adenoid cystic carcinoma; EGC, esophageal cancer; GC, gastric cancer; SCLC, small cell lung cancer.

**Figure S2. Maximum Percentage Changes From Baseline in Sums of Diameters of Target Lesions**


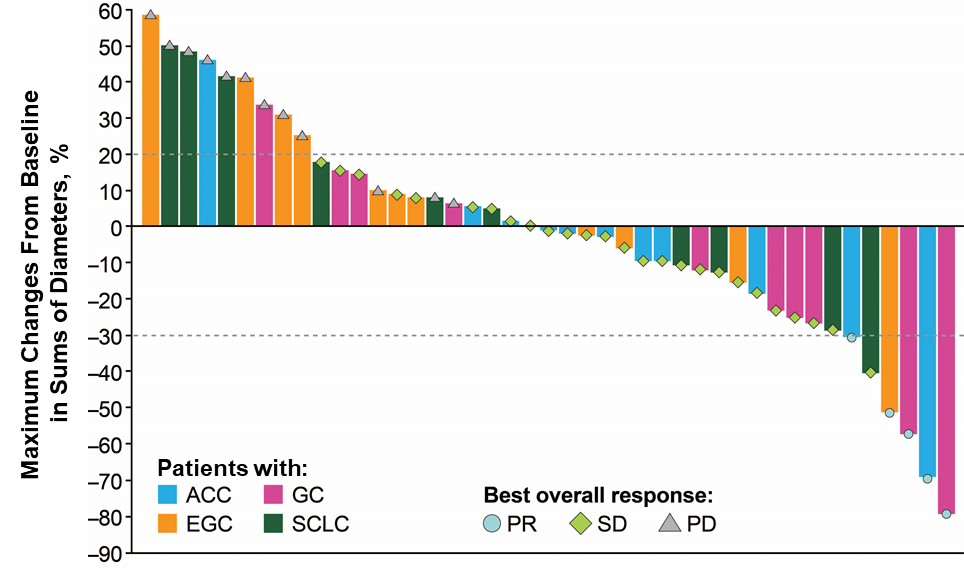
ACC, adenoid cystic carcinoma; EGC, esophageal cancer; GC, gastric cancer; PD, progressive disease; PR, partial response; SCLC, small cell lung cancer; SD, stable disease.
